# The role of individual differences in emotional word recognition: Insights from a large-scale lexical decision study

**DOI:** 10.3758/s13428-024-02488-z

**Published:** 2024-09-04

**Authors:** Juan Haro, José Antonio Hinojosa, Pilar Ferré

**Affiliations:** 1 Departament de Psicologia and CRAMC, Universitat Rovira i Virgili, Carretera de Valls, s.n., 43007 Tarragona, Spain; 2 Departamento de Psicología Experimental, Procesos Cognitivos y Logopedia, Universidad Complutense de Madrid, Madrid, Spain; 3 Instituto Pluridisciplinar, Universidad Complutense de Madrid, Madrid, Spain; 4 Centro de Investigación Nebrija en Cognición (CINC), Universidad Nebrija, Madrid, Spain

**Keywords:** Emotional valence, Word recognition, Individual differences, Personality traits, Lexical decision task

## Abstract

This work presents a large lexical decision mega-study in Spanish, with 918 participants and 7500 words, focusing on emotional content and individual differences. The main objective was to investigate how emotional valence and arousal influence word recognition, controlling for a large number of confounding variables. In addition, as a unique contribution, the study examined the modulation of these effects by individual differences. Results indicated a significant effect of valence and arousal on lexical decision times, with an interaction between these variables. A linear effect of valence was observed, with slower recognition times for negative words and faster recognition times for positive words. In addition, arousal showed opposite effects in positive and negative words. Importantly, the effect of emotional variables was affected by personality traits (extroversion, conscientiousness and openness to experience), age and gender, challenging the 'one-size-fits-all' interpretation of emotional word processing. All data collected in the study is available to the research community: https://osf.io/cbtqy. This includes data from each participant (RTs, errors and individual differences scores), as well as values of concreteness (*n* = 1690), familiarity (*n* = 1693) and age of acquisition (*n* = 2171) of the words collected exclusively for this study. This is a useful resource for researchers interested not only in emotional word processing, but also in lexical processing in general and the influence of individual differences.

## Introduction

The study of the processes that come into play in word recognition has been one of the central topics in psycholinguistics, revealing some of the cognitive mechanisms involved in the access to word form and meaning (Balota et al., 2006). This research interest has also provided insights into how lexical content is structured and represented in the mind and has stimulated the development of word recognition computational models (e.g. Marslen-Wilson & Welsh, 1978; McClelland & Rumelhart, 1981; see Norris, 2013, for a review).

One of the most widely used tasks to study lexical processing and representation is the Lexical Decision Task (LDT; Rubenstein et al., 1970), in which participants are asked to decide whether a string of letters is a word or a pseudoword. Although the LDT is a shallow task, there is considerable evidence of the influence of several lexical variables. For example, reaction times (RTs) and accuracy in the LDT are modulated by, but not limited to, objective and subjective lexical frequency (e.g. Balota & Chumbley, 1984; Balota et al., 2004), word length (e.g. New et al., 2006), number of orthographic (e.g. Pollatsek et al., 1999) and phonological (e.g. Luce & Pisoni, 1998) neighbours, syllable frequency (Macizo & Van Petten, 2007), bigram frequency (e.g. Westbury & Buchanan, 2002), average Levenshtein distance of the 20 nearest lexical neighbours (OLD20; Yarkoni et al., 2008), and contextual diversity (e.g. Adelman et al., 2006; Johns, 2021). Remarkably, there is also evidence that semantic variables influence LDT responses. This suggests the involvement of a top-down mechanism during word recognition, i.e., before the meaning of the word is accessible, the semantic representation of the word is partially activated and affects the processing of the orthographic input (Balota, 1990). For instance, research has revealed effects of word concreteness (James, 1975), number of semantic features (Pexman et al., 2002), number of meanings (Haro & Ferré, 2018), number of lexical (Buchanan et al., 2001) and semantic associates (Mirman & Magnuson, 2008), sensory experience (Juhasz et al., 2011), among others. All these variables contribute to so-called semantic richness, a multidimensional construct referring to the quantity and diversity of semantic information associated with a word (Pexman et al., 2008; Yap et al., 2015). Research has demonstrated how semantic richness facilitates word recognition, possibly through semantic-to-orthographic feedback mechanisms (see Yap et al., 2015).

A variable that has been included within the semantic richness construct is the emotional content of words (e.g. Goh et al., 2016; Yap & Seow, 2014). It has been extensively studied over the last decades, showing a reliable influence in word recognition (see Citron, 2012; Hinojosa et al., 2020, for reviews). The emotional content of a word is usually defined in terms of continuous variations of two main affective dimensions: emotional valence and emotional arousal (Bradley & Lang, 1999). Valence represents the hedonic value of the emotional response evoked by the word, ranging from very unpleasant/negative to very pleasant/positive, whereas arousal refers to the activation associated to that emotional response, ranging from very relaxing to very activating.

While there is evidence that both valence and arousal affect word recognition, valence appears to have a stronger effect than arousal (Kousta et al., 2009; Kuperman et al., 2014). However, there is no consensus regarding the direction of the effects of these two variables. With respect to arousal, some studies have found a facilitation effect (faster RTs for high arousal words than for low arousal words, e.g. Estes & Adelman, 2008), while others have obtained an inhibition effect (Kuperman et al., 2014) or even a null effect of this variable (Kousta et al., 2009; Rodríguez-Ferreiro & Davies, 2019). A similar or even more complex picture emerges for valence effects. Experimental evidence largely points to a facilitation for positive words (i.e., faster RTs) compared to neutral and negative words (e.g. Kuchinke et al., 2005; Rodríguez-Ferreiro & Davies, 2019; Siakaluk et al., 2016; but see Bayer et al., 2012). In contrast, the effects of negative valence are mixed. Some studies have found a facilitation for negative words relative to neutral words (Citron et al., 2013; Kuchinke et al., 2007; Vinson et al., 2014); others have reported an inhibition (i.e., slower RTs for negative words than for neutral words, e.g. Estes & Adelman, 2008; Larsen et al., 2008; Yao et al., 2016), and yet others have found no significant effects of negative valence (Kuchinke et al., 2005; Larsen et al., 2006). This is further complicated by the fact that valence and arousal can interact during word recognition. Indeed, high arousal facilitates the recognition of negative words and, conversely, inhibits the recognition of positive words (e.g. Citron et al., 2014; Hofmann et al., 2009; Larsen et al., 2008; Vieitez et al., 2021; but see Kuperman et al., 2014).

The inconsistencies observed in emotional word recognition research may be due to a lack of experimental control. In their review of studies using the emotional Stroop task and the LDT, Larsen et al. (2006) found that negative words in these studies were typically longer, less frequent, and had fewer lexical neighbours than their positive and neutral counterparts. Furthermore, Warriner et al. (2013) showed that positive words tend to be more concrete, familiar, contextually rich, and acquired earlier than both neutral and negative words. Given the myriads of lexical and semantic variables that influence LDT, rigorous experimental control is essential for a refined understanding of emotional word recognition and, by extension, word recognition in general. This need for experimental control has led to a proliferation of lexical decision mega-studies over the last two decades (see Keuleers & Balota, 2015). In these studies, RTs are collected for many words from large numbers of participants, while controlling for the effect of multiple variables that influence word recognition. Since the publication of the first LDT mega-study in English by Balota et al. (2004, see also Balota et al., 2007), this approach has been used in several languages, such as French (Ferrand et al., 2010), Chinese (Tse et al., 2016), Dutch (Keuleers et al., 2010), German (Schröter & Schroeder, 2017), Malay (Yap et al., 2010), Korean (Yi et al., 2017), Portuguese (Soares et al., 2019) and Spanish (González-Nosti et al., 2014).

Mega-studies offer a number of advantages over conventional LDT studies. A main advantage is that they provide the possibility to test the effect of continuous variables over their full range without the need for discretisation. This approach avoids the limitations of factorial designs, which often restrict the selection of stimuli to words that vary in one or a few dimensions. Mega-studies also provide greater control over potential confounding factors. They take advantage of the ability of linear mixed-effects models to account for the random variance associated with subjects and items. Mega-studies also minimise the influence of experimental lists, of biases in stimulus selection, and of the impact of the researcher's implicit knowledge in stimulus selection (Forster, 2000). Last but not least, mega-studies allow broader inferences to be made about lexical processing, rather than being restricted to a particular set of words (e.g. conducting virtual experiments; see Kuperman, 2015).

The aim of this work was to conduct a LDT mega-study involving a large set of Spanish words, with a primary interest in the effects of emotional content on visual word recognition. A few mega-studies have focused on emotional word recognition so far. In general, the results indicate a linear relationship between emotional valence and RTs: positive words speed up recognition compared to neutral and negative ones, while negative words slow it down (e.g. Kuperman, 2015; Kuperman et al., 2014; Rodríguez-Ferreiro & Davies, 2019). Some studies, however, have reported an inverted U-shaped valence effect, with both positive and negative words being responded to faster than neutral words (Vinson et al., 2014). As for the effect of arousal, the results are mixed. While Vinson et al. (2014) and Rodriguez-Ferreiro and Davies (2019) did not observe a significant arousal effect, Estes and Adelman (2008) and Kuperman et al. (2014) did. Notably, the results of the last two studies were polarized: Estes and Adelman found a facilitative effect for arousal, whereas Kuperman et al. found an inhibitory effect.

One issue that has not been considered in the above-mentioned mega-studies is the role of individual differences, which may contribute to the inconsistent findings in the field. Individual differences in language processing are pervasive (Kidd et al., 2018), and they are observed across all domains, like word recognition (Yap et al., 2012), reading (Kuperman & Van Dyke, 2011) or syntactic processing (Wells et al., 2009), just to name a few. Other cognitive functions also affect performance in linguistic tasks, such as working memory capacity (e.g. Conti‐Ramsden et al., 2001; Just & Carpenter, 1992) and executive function (e.g. Novick et al., 2014; Nozari et al., 2016). Affective-motivational factors influence language processing as well (see Fox, 2020). The study of those extra-linguistic influences can reveal highly valuable information about language comprehension processes (Hubert Lyall, 2019). However, psycholinguistics research has traditionally neglected them, by averaging over individual differences and extra-linguistic information to make inferences about the general population. This has started to change in the last years, with an emerging interest for these topics (see Kidd et al., 2018, and Fox, 2020).

A main novelty of this mega-study is that we examined the role of individual differences on several variables on the emotional effects in visual word recognition, focusing on personality traits, gender, and age. Studies examining the role of personality traits in emotional processing have mostly relied on images/pictures (e.g. De Young et al., 2010; Kehoe et al., 2012), and research focusing on linguistic stimuli is scarce. The usual approach here has been to collect data on various personality characteristics belonging to the participants through self-report questionnaires and examine whether they modulate the processing of the affective content of the stimuli (i.e., valence and/or arousal). For instance, Silva et al. (2012) found that the effect of negative valence in a LDT was modulated by participants’ disgust sensitivity: Only participants who had high disgust-sensitivity showed slower RT to negative disgusting words than to neutral words. Other studies have focused on the Big Five personality traits (Goldberg, 1990): extroversion (sociability and energy; from introverted to extroverted), openness to experience (creativity and willingness to try new experiences; from conservative to innovative), agreeableness (empathy and cooperation; from challenging to cooperative), emotional stability (ability to handle stress; from neurotic to emotionally stable) and conscientiousness (organization and reliability; from impulsive to methodical). In this line, Borkenau et al. (2010) found that the higher the level of extroversion of participants, the faster the recognition of positive words compared to neutral or negative words. This is consistent with the idea that extroverts, with respect to introverts, are more sensitive to positive cues and that pleasant concepts are more accessible to them. Similarly, Ku et al. (2020) observed that both extroversion and neuroticism influenced emotional word recognition in a LDT where event-related potentials (ERP) were recorded. In contrast, Johnson et al. (2023) failed to find a clear effect of either extroversion or neuroticism in reading emotion words embedded in sentences, although high scores in agreeableness and openness to experience were related to a facilitated processing for negative words. Other personality traits have been shown to influence emotional word processing in the LDT, for instance goal-directed behaviour (Mueller & Kuchinke, 2016) and the degree of acceptance of epistemically unwarranted beliefs (Huete-Pérez & Ferré, 2022). In addition, individual differences in emotion regulation skills have been related with performance in the LDT, with individuals with higher capacity exhibiting faster responses to positive words than to negative words (Taroyan et al., 2020). In contrast, a high degree of need for affect has been shown to increase reading times for positive words embedded in a story (Lei et al., 2023). Overall, these findings evidence the influence of several personality traits in emotional word recognition, a role that has been also acknowledged in studies focused on language use. For instance, Yarkoni (2010) examined the association between personality and word use in a large sample of texts from web blogs and found that high neuroticism was associated with a greater use of negative words while extroversion and agreeableness were associated with a greater use of positive words. The association between extroversion and the use of positive emotion words (as well as social-related words) has been confirmed in a recent meta-analytic study (Chen et al., 2020). Furthermore, openness to experience is usually marked by the elevated use of insight-related words and conscientiousness by the low use of negative emotion words (Caplan et al., 2020).

Gender has been pointed to as another modulator factor of the emotional response to stimuli, with some studies evidencing a more intense emotional reaction in females compared to males (e.g. Bianchin & Angrilli, 2012; Tobin et al., 2000). This has led some authors to investigate if gender effects exist in the response to affective word content as well. Most studies in this line have relied on affective ratings. As an example, Bauer and Altarriba (2008) found that females rated concrete words as more emotional than males. The results of Soares et al. (2012) and Söderholm et al. (2013) went in the same direction: Females provided more extreme valence ratings (i.e., they perceived positive words as more positive and negative words as more negative) and higher arousal ratings than males. In contrast, other authors have failed to find any difference between males and females in the affective ratings of words (Gilet et al., 2012; Redondo et al., 2007). Differences between genders have also been observed in children, with studies showing higher valence scores in girls (Sylvester et al., 2016) or, conversely, in boys (Sabater et al., 2020). Another line of research has focused on word use. For instance, Sells and Randal (2001) recorded participants’ responses after watching an emotionally eliciting video and found that women used more emotional words than men. Consistent with that, female participants produced more words in an emotional semantic fluency task (i.e., participants were asked to produce as many words referred to emotions as they could) compared to males (Yeung, 2023). Of note, despite the above evidence suggesting gender effects in the response to emotional words and in emotional expressivity, this topic has hardly been investigated so far in the field of emotional word recognition, and those few studies yielded mixed findings. Indeed, Abbassi et al. (2019) showed that females processed emotional words faster than males in a divided visual field affective priming study. In contrast, Naranowicz et al. (2023) did not obtain a gender effect in an affective decision task, in which participants were asked to decide whether a set of words were positive, negative or neutral.

A last variable worth mentioning here with regards to emotional word processing is age. A general positivity bias has been described in older people (that is, a preferential processing for positive over negative information; see Carstensen & DeLiema, 2018, for an overview). Although many studies have focused on pictorial stimuli (e.g. Meng et al., 2015; Steenhaut et al., 2018), research investigating whether this positivity bias extends to linguistic stimuli is increasing. Accordingly, some affective rating studies have shown that older people give more positive ratings to words than younger people (e.g. Grandy et al., 2020). However, findings are mixed, with some studies showing that older people provide more positive ratings for positive words and less arousing ratings for negative words than younger people (Grühn & Smith, 2008, Liu et al., 2021), while in other studies older people rated negative words as more arousing than younger people (Ready et al., 2017). Research relying on word processing tasks has led to inconclusive results too. Indeed, Ku et al. (2022) confirmed the aging positivity bias in a LDT where ERP were recorded, while Allegretta et al. (2021) did not find any effect of emotional content of words embedded in sentences in older people. As Kyröläinen et al. (2021) pointed out, one limitation of previous studies investigating the effects of age on emotional word recognition was the small number of participants and the use of a dichotomous design, in which two groups of participants (young and old) are compared. This experimental design cannot shed light on whether the change in the emotional response develops gradually. Kyröläinen et al. overcame this limitation by retrieving data from three published large-scale LDT studies, which included a wide range of age between their participants. They found a decrease in the positivity bias with age, contrary to what should be expected from the proposed preferential processing of positive over negative information (e.g. Carstensen & DeLiema, 2018).

Considering the studies reviewed in the previous paragraphs, there is solid evidence of the influence of emotional content on visual word recognition. However, results are inconclusive regarding the direction of the effects. The reason for these contradictory findings is unclear. One possibility is that some studies have not considered the role of the lexico-semantic variables related with the words that influence their processing. Another, and much less investigated, reason may be that they have ignored the presence of individual differences in the processing of emotional information, and that the presence of those differences has affected the outcome of these studies. To overcome these limitations, we conducted a large scale LDT study, involving 7500 Spanish words, which were characterised in many variables that are known to affect word processing. Furthermore, we investigated the modulation of these effects by individual differences in several personality traits and demographic variables which have been proposed to affect the response to emotional stimuli: the Big Five personality traits, age, and gender. The contribution of this study is two-fold. On the one hand, it may provide very relevant information concerning the role of emotional content in visual word processing and its modulation by individual variables. This information should be considered by theoretical models, which have traditionally neglected these topics (Fox, 2020; Kidd et al., 2018). On the other hand, we make available a dataset including RT and error data for many words characterized in a large set of variables, which can be used by researchers in the field to test their hypotheses about visual word recognition.

## Method

### Participants

A total of 918 native Spanish speakers (mean age = 27.51, range = 17–70, SD = 11.05; 69.83% female) completed the study. They were recruited through various online channels, including social media posts, mailing lists, emails to the university community, and WhatsApp messages. They received monetary compensation for their participation. All participants had normal or corrected-to-normal vision; 91.2% were right-handed, 7.6% were left-handed and 1.2% were ambidextrous. They signed an informed consent form before participating in the study. The study was approved by the Ethics Committee of the University of Rovira i Virgili (reference CEIPSA-2021-PR-0044).

### Materials

We selected a total of 7500 Spanish words for the experiment: 5235 nouns (69.80%), 1252 verbs (16.69%) and 1013 adjectives (13.51%). They comprised a wide range of values for several variables known to influence LDT: emotional valence, emotional arousal, concreteness, familiarity, age of acquisition, logarithm of frequency, number of letters, number of syllables, number of phonemes, number of lexical neighbours, number of higher-frequency lexical neighbours, average Levenshtein distance of the 20 nearest lexical neighbours (OLD20), number of phonological neighbours, number of higher-frequency phonological neighbours, average bigram frequency, and average trigram frequency (see Table 1 for descriptive statistics).

**Table 1:** Descriptive statistics of the words used in the experiment

Variable	Mean	SD	Min	Max
Valence	5.24	1.49	1.15	8.85
Arousal	5.37	1.03	1.40	8.45
Concreteness	4.61	1.04	1.30	6.95
Familiarity	5.12	1.03	1.40	7.00
Age of acquisition	7.44	2.00	1.70	11.00
Log. frequency	0.87	0.61	0.00	3.95
Letters	7.56	2.27	2.00	17.00
Lexical neighbours (N)	3.63	5.49	0.00	40.00
Higher-frequency N	0.54	1.45	0.00	22.00
OLD20	2.05	0.68	1.00	5.55
Bigram frequency	26258	10683	15.30	71639
Trigram frequency	2916	2385	0.58	19937
Phonemes	7.43	2.30	2.00	17.00
Syllables	3.14	0.95	1.00	7.00
Phonological neighbours (PN)	7.90	10.60	0.00	91.00
Higher-frequency PN	1.04	2.43	0.00	33.00

The values for these variables were taken from several normative sources. Values for emotional valence and arousal were obtained from Ferré et al. (2012), Guasch et al. (2016), Hinojosa et al. (2016a, 2016b), and Stadthagen-González et al. (2018). Familiarity and concreteness ratings were retrieved from Ferré et al. (2012), Guasch et al. (2016), Hinojosa et al. (2016a, 2016b), Huete-Pérez et al. (2019), and ESPAL (Duchon et al., 2013). Age of acquisition values were obtained from Alonso et al. (2015), Hinojosa et al. (2016a, 2016b), and Huete-Pérez et al. (2019). The values for the lexical and phonological variables were taken from ESPAL (Duchon et al., 2013), an online repository of Spanish word properties (http://www.bcbl.eu/databases/espal/). Of note, the word frequency values obtained from EsPal are derived from Spanish subtitles of films and TV series.

There were no normative data for some words in age of acquisition (*n* = 2171), concreteness (*n* = 1690), and familiarity (*n* = 1693). These values were obtained through ad hoc questionnaires created with TestMaker (Haro, 2012). The participants who rated the words were undergraduate students from the Universitat Rovira i Virgili (Tarragona, Spain), who received a bonus of academic credits for their participation. They all signed an informed consent form prior to the study. A sample of 428 participants rated the words in terms of age of acquisition (mean age = 20 years, SD = 4; range = 18–59; 87% female); 467 participants rated the words in terms of concreteness (mean age = 20 years, SD = 4; range = 18–59; 88% female) and 467 participants rated the words in terms of familiarity (mean age = 20 years, SD = 4; range = 18–59; 88% female). There were 19 versions of the questionnaire for age of acquisition (with either 100, 120 or 180 words; mean words rated by each participant = 128, SD = 20), 18 versions for concreteness (with either 100, 140 or 180 words; mean words rated by each participant = 120, SD = 34), and 18 versions for familiarity (with either 100, 140 or 180 words; mean words rated by each participant = 123, SD = 30). Each word was rated for age of acquisition, concreteness and familiarity by at least 25 participants. However, because participants could indicate that they did not know the word, not all words received at least 25 valid ratings (age of acquisition: mean valid ratings = 23, SD = 4; concreteness: mean valid ratings = 24, SD = 4; familiarity: mean valid ratings = 25, SD = 4).

We used the same instructions and scales for the rated variables as other Spanish normative studies: age of acquisition (Alonso et al., 2015; Hinojosa et al., 2016a, 2016b; Huete-Pérez et al., 2019), concreteness (Duchon et al., 2013; Guasch et al., 2016) and familiarity (Duchon et al., 2013; Guasch et al., 2016). In this way, our values can be directly compared with those reported in these databases. The full instructions are available as supplementary material in the OSF online repository (see “Appendix A”).

Participants' responses were examined to assess the reliability of the data. We removed data from participants whose ratings showed a low correlation with the mean ratings of all participants completing the same version of the questionnaire (i.e., r < 0.1; in line with similar normative studies, e.g. Haro et al., 2017). Correlation values close to zero were interpreted as idiosyncratic response patterns, while negative values would indicate that the participant understood the scale in reverse order. We also removed data from participants who completed the same questionnaire twice, as well as data from participants who responded to less than 50% of the words. This process led us to exclude the responses of seven participants from the age of acquisition questionnaires, 37 from the concreteness questionnaires and ten from the familiarity questionnaires. We also calculated the mean intraclass correlation coefficients (ICCs) of the questionnaires to assess the interrater reliability of the variables. All variables showed an excellent reliability (see Cicchetti, 1994, for interpretation guidelines): mean = .98 for age of acquisition (SD = .01; range = .95 to .99), mean = .97 for concreteness (SD = .01; range = .94 to .98), and mean = .99 for familiarity (SD = .00; range = .98 to .99).

Once we had collected all the values for the relevant variables, we created the experimental lists. The full set of words was divided into 25 lists of 300 words each. Kruskal–Wallis test was used to ensure that the distribution of the values of the considered variables was the same across the lists (all *p*s > .169). We also created 7500 pseudowords using the Wuggy software (Keuleers & Brysbaert, 2010), based on the selected words. Some pseudowords were adapted: accents were added, and endings were changed if they were rare or illegal in Spanish. Each list included the corresponding 300 pseudowords generated from the 300 words.

Two questionnaires were used to collect information from the participants. An ad hoc socio-demographic questionnaire was created to obtain age, gender, native language, visual acuity (normal or corrected), dominant hand, history of neurological disease, psychological disorders, and alcohol or drug abuse. In addition, the Overall Personality Assessment Scale (OPERAS-40; Vigil-Colet et al., 2013) was used to measure the five-factor model of personality traits (i.e., extroversion, openness to experience, agreeableness, emotional stability and conscientiousness). It is a short scale that includes 40 questions with responses ranging from 'strongly disagree' (1) to 'strongly agree' (5).

### Procedure

Before beginning the experimental task, participants signed an informed consent document and completed the socio-demographic questionnaire. Each participant was then randomly assigned to one of the 25 stimulus lists. Participants completed a LDT consisting of 600 trials. The task was presented using JsPsych (de Leeuw, 2015). Each trial began with a fixation dot displayed for 500 ms. This was followed by a stimulus, which could either be a word or a pseudoword, presented for up to 2000 ms or until the participant responded. Participants had to press the 'J' key for the 'yes' response (if they identified the stimulus as a real Spanish word) and the 'F' key for the 'no' response (if they considered that the stimulus was not a real Spanish word). The stimuli appeared in a random order for each participant and no feedback was given. The task was administered automatically: as soon as the participant responded, or when the 2000 ms time-limit expired, the next trial automatically began, with an intertrial interval of 750 ms. There were 20 practice trials at the beginning of the task, during which feedback was provided. There were two breaks, one every 200 trials. The task was administered online. Participants were required to access the website where the experiment was hosted. It could only be done on a PC; mobile devices, tablets, etc., were not allowed.

After completing the LDT, participants were asked to fulfil the OPERAS-40 questionnaire. Only participants who completed both the LDT and the questionnaire were included in the study. The experimental session lasted around 45 min. The data collected was completely anonymous.

### Data analyses

We excluded the data from participants with more than a 15% error rate (*n* = 59) and from words with an error rate above 75% (*n* = 5). After this, the mean number of observations for each of the remaining 7495 words was 36.72 (range: 31–43, SD = 2.66). We also discarded all observations with RTs below 300 ms from the analyses, as well as those that were 2.5 standard deviations above or below the mean RT for each participant. The overall accuracy was high (mean = 94.73%), so we decided not to analyse the error data. In total, we removed 50,360 observations (8.59% of the total), leaving 506,848 observations for analysis. All this data (RTs and errors), as well as that given by the participants (personality trait scores, age and gender) and the values retrieved from the words (psycholinguistic scores), are available in the online repository: https://osf.io/cbtqy.

We analysed the data with linear mixed effects models (e.g. Baayen, 2008; Baayen et al., 2008). We used the lme4 package in R (Bates et al., 2015). A model was generated to examine the main effects and interactions of the variables of interest on raw RTs^1^. Emotional valence, emotional arousal, and their interactions with personality trait scores (extroversion, openness to experience, agreeableness, emotional stability and conscientiousness), age, and gender were introduced as fixed effects. We also included several covariates known to influence LDT (see Table 1 and “Appendix B” in the OSF repository). All covariates were centred and transformed into *z*-scores. As we did not find a three-way interaction between valence and arousal and each of the fixed effects of interest, only the second-order interactions were included in the model. Random intercepts were included for participants and words; it was not possible to add random slopes due to convergence issues. We calculated multicollinearity between fixed effects (R VIF function) and removed those with a VIF > 5 or with a correlation > .80 with other fixed effects; in particular, we removed number of phonemes, number of phonological neighbours, number of higher frequency phonological neighbours, and number of syllables^2^. Participant gender was coded using sum contrast coding: female (– 0.5), male (+ 0.5). The full formula of the model is shown below:

$$\text{RT }\sim \text{valence}\times\text{arousal }+\text{ valence}\times\text{emotional stability }+\text{ valence}\times \text{agreeableness }+\text{ valence}\times \text{extroversion }+\text{ valence}\times \text{openness to experience }+\text{valence}\times \text{conscientiousness }+\text{ valence}\times \text{age }+\text{ valence}\times \text{gender }+\text{ arousal}\times \text{emotional stability }+\text{ arousal}\times \text{agreeableness }+\text{ arousal}\times\text{extroversion }+\text{arousal}\times \text{openness to experience}+\text{ arousal}\times \text{conscientiousness }+\text{ arousal}\times \text{age }+\text{ arousal}\times \text{gender }+\text{concreteness }+\text{ familiarity }+\text{ age of acquisition }+\text{ log frequency }+\text{ letters }+\text{number of neighbours }+\text{number of higher frequency neighbours }+\text{old}20 +\text{bigram frequency }+\text{trigram frequency }+\text{trial order }+ (1|\text{ participant}) + (1 |\text{word})$$

In addition, we created a second model, identical to the previous one, but replacing the continuous valence variable by a categorical variable. Categorical valence was created by dividing continuous valence into three categories: negative valence (valence range: 1–3.49), neutral valence (valence range: 3.5–5.49), and positive valence (valence range: 5.5–9). This model was designed to examine the effects of negative and positive valence in relation to neutral (i.e., non-emotional) words. Unless otherwise stated, the results presented in the Results section are those from the continuous valence model. Results from the categorical valence model are shown to give a more accurate picture of the meaning of the main effects and interactions observed in the continuous valence model.

Multiple comparisons were conducted using the emmeans software package (Lenth, 2020). For continuous variables, marginal means were calculated at two standard deviations below (hereafter 'low level') and above (hereafter 'high level') the mean, and at the mean itself (hereafter ‘medium level'). These levels were used for multiple comparisons of continuous variables, and also for plots showing the interactive effects where there was at least one continuous variable involved. Multiple comparisons were corrected using the Tukey method. We also report the results of *t* test analyses of the coefficient estimates for each fixed effect and interaction. For this purpose, we used Satterthwaite's approximation to the degrees of freedom of the denominator (p values were estimated using the lmerTest package, Kuznetsova et al., 2017).

## Results

**Fig. 1:**
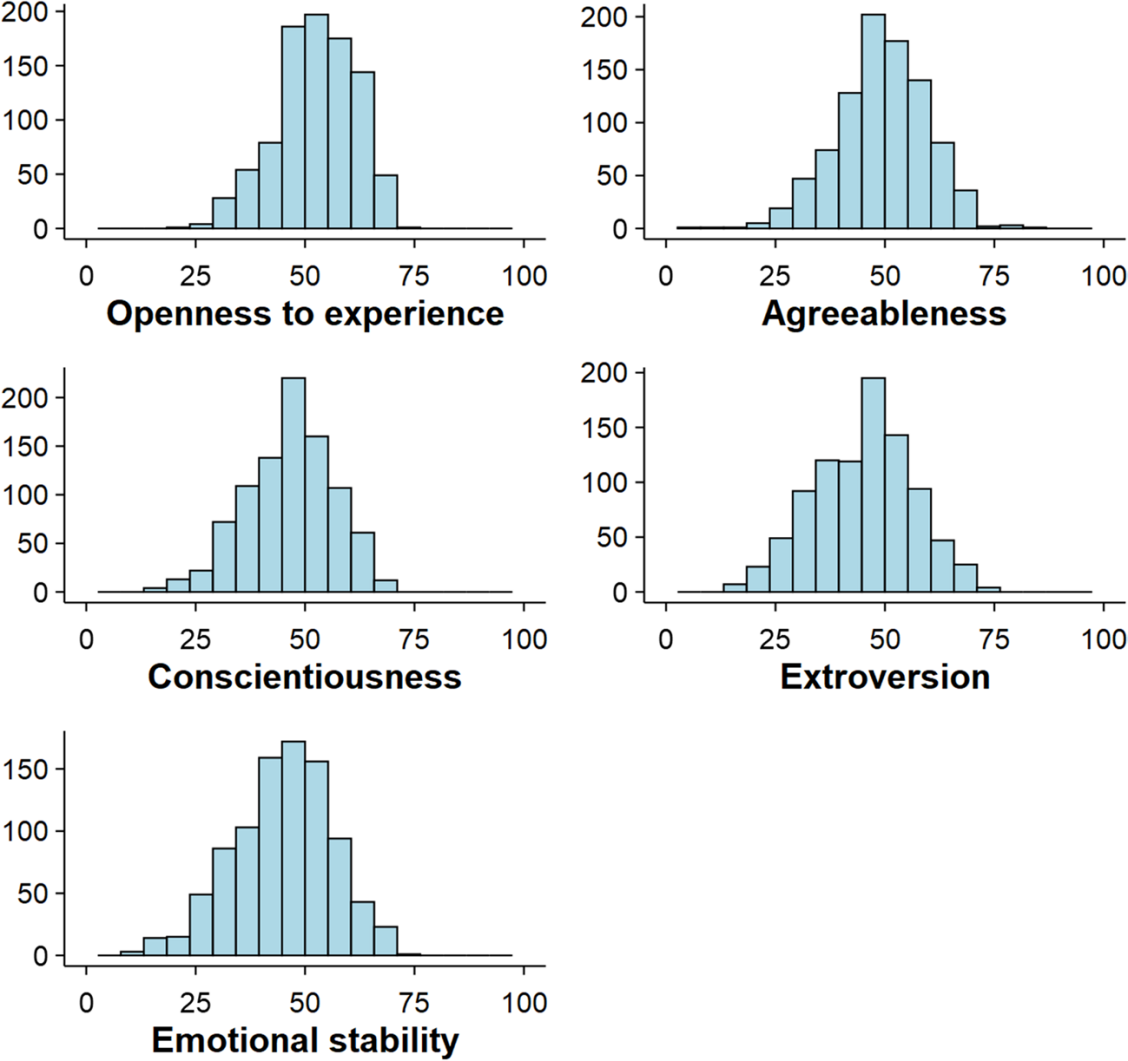
Distribution of personality trait scores

**Fig. 2:**
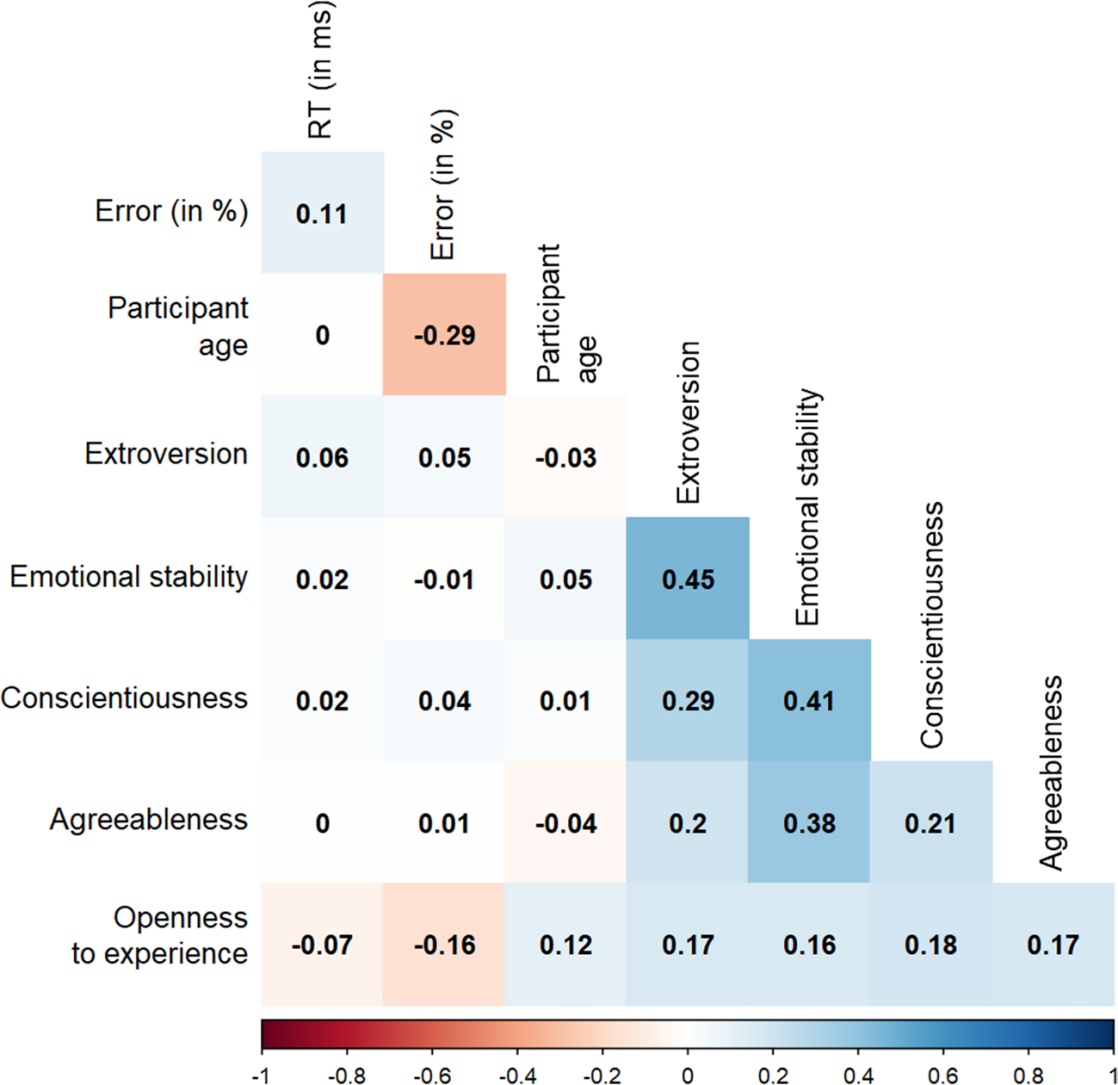
Correlogram between participants’ measures

Table 2 shows the descriptive statistics of the participant related variables. Figure 1 shows the distribution of the participants' scores on the five personality traits, obtained from the OPERAS-40 questionnaire. The personality scores were relatively normally distributed, with means ranging from 44.11 to 54.04, standard deviations ranging from 8.82 to 11.40 points, and presenting hardly any extreme scores. In addition, Pearson correlations were computed between all the measures obtained from the participants (see Fig. 2).

**Table 2:** Descriptive statistics of the variables related to the participants and of their performance in the LDT

Variable	Mean	SD	Min	Max
LDT RT	750.51	177.99	373.90	1414.20
LDT %E	5.29	3.13	0.33	15.00
Extroversion	46.49	11.40	15.00	81.00
Emotional stability	44.11	11.14	10.00	69.00
Conscientiousness	46.61	10.06	10.00	74.00
Agreeableness	51.33	10.32	8.00	82.00
Openness to experience	54.04	8.82	18.00	71.00

The results of the linear mixed effects model (the one that included valence as a continuous variable) revealed a number of interactions and main effects (see Table 3). These interactions and main effects are described below, along with the results of multiple comparisons where appropriate. As indicated in the “Data analyses” section, the results of the continuous valence model are in some cases complemented by the results of the categorical valence model.

**Table 3:** Results of the linear mixed effect model (continuous valence)

Predictors	Estimate	SE	CI	*t*	*P*
*Intercept*	650.68	3.38	644.04–657.31	192.29	**< 0.001**
Valence	– 5.68	0.66	– 6.98 to – 4.38	– 8.58	**< 0.001**
Arousal	– 2.39	0.60	– 3.57 to – 1.22	– 4.01	**< 0.001**
Emotional stability	1.45	3.89	– 6.17 to 9.07	0.37	0.709
Agreeableness	– 1.22	3.34	– 7.77–5.33	– 0.37	0.714
Extroversion	10.79	3.47	3.99–17.59	3.11	**0.002**
Openness to experience	– 13.34	3.16	– 19.54 to – 7.14	– 4.22	**< 0.001**
Conscientiousness	0.27	3.44	– 6.46 to 7.00	0.08	0.938
Participant age (years)	– 0.14	3.14	– 6.29 to 6.01	– 0.05	0.964
Participant gender	0.26	6.99	– 13.44 to 13.97	0.04	0.970
Concreteness	2.85	0.55	1.77–3.94	5.14	**< 0.001**
Familiarity	– 17.17	0.61	– 18.38 to – 15.97	– 27.99	**< 0.001**
Age of acquisition	9.45	0.70	8.09–10.82	13.56	**< 0.001**
Log. Frequency	– 19.88	0.69	– 21.22 to – 18.53	– 28.95	**< 0.001**
Letters	0.89	0.90	– 0.86 to 2.65	1.00	0.319
Lexical neighbours	7.27	0.84	5.62–8.93	8.61	**< 0.001**
Higher lexical neighbours	– 0.17	0.71	– 1.55 to 1.21	– 0.24	0.812
OLD20	10.03	0.89	8.29–11.77	11.30	**< 0.001**
Bigram frequency	5.26	0.71	3.87–6.64	7.45	**< 0.001**
Trigram frequency	2.28	0.71	0.89–3.68	3.21	**0.001**
Trial order	2.34	0.27	1.81–2.88	8.61	**< 0.001**
Valence * Arousal	2.36	0.44	1.49–3.22	5.35	**< 0.001**
Valence * Agreeableness	– 0.33	0.35	– 1.01–0.35	– 0.94	0.345
Valence * Conscientiousness	– 0.79	0.36	– 1.49 to – 0.10	– 2.24	**0.025**
Valence * Emotional stability	– 0.35	0.40	– 1.13–0.44	– 0.86	0.389
Valence * Extroversion	– 0.95	0.36	– 1.66 to – 0.24	– 2.63	**0.008**
Valence * Openness to experience	1.51	0.33	0.86–2.16	4.55	**< 0.001**
Valence * Participant age (years)	0.81	0.33	0.16–1.45	2.45	**0.014**
Valence * Participant gender	3.24	0.72	1.82–4.66	4.49	**< 0.001**
Arousal * Agreeableness	– 0.41	0.35	– 1.09–0.27	– 1.19	0.236
Arousal * Conscientiousness	0.14	0.35	– 0.55–0.84	0.40	0.692
Arousal * Emotional stability	0.39	0.40	– 0.40–1.18	0.96	0.337
Arousal * Extroversion	– 0.19	0.36	– 0.89–0.52	– 0.52	0.606
Arousal * Openness to experience	0.76	0.33	0.11–1.41	2.30	**0.021**
Arousal * Participant age (years)	1.22	0.33	0.57–1.87	3.69	**< 0.001**
Arousal * Participant gender	– 1.26	0.72	– 2.68–0.16	– 1.74	0.081

### Valence and arousal

**Fig. 3:**
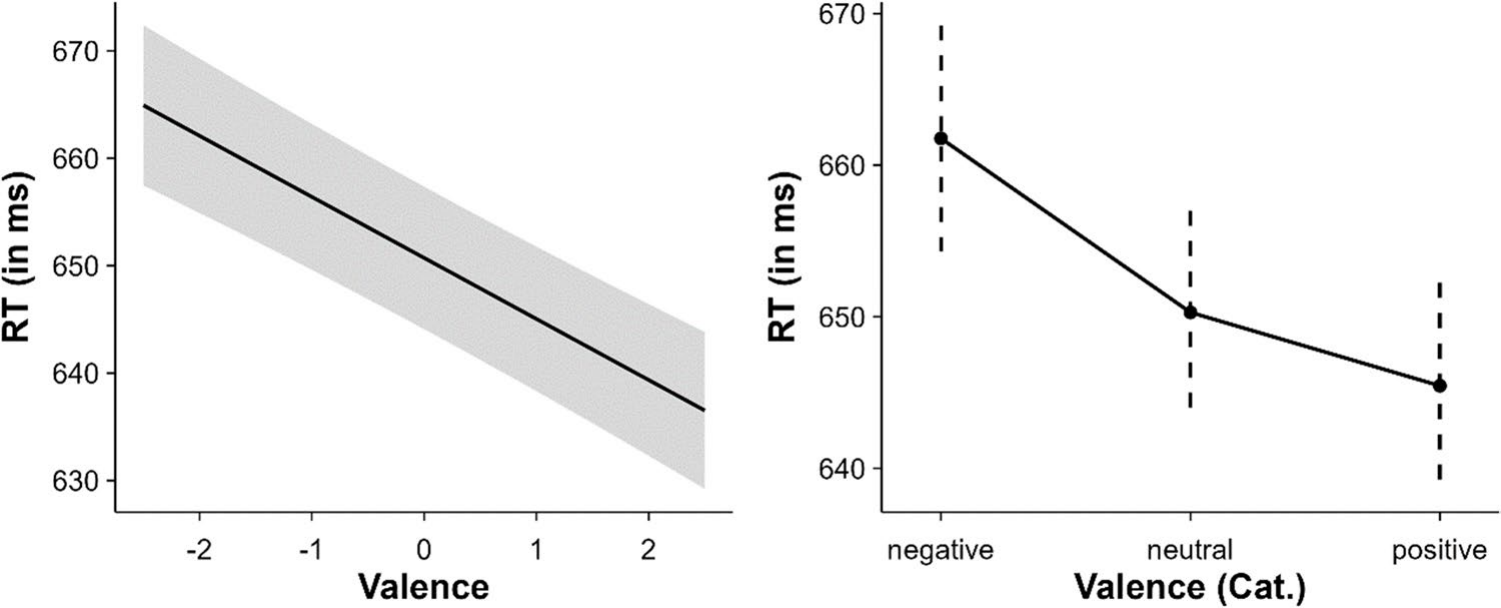
Effect of valence (*left* = continuous valence model; *right* = categorical valence model)

A significant effect of valence was observed, *β* = – 5.68, SE = 0.66, *t* = – 8.58, *p* < .001. This effect indicates that, as valence increases, RT decreases. That is, positive words were recognised faster than neutral and negative words, and negative words were recognised slower than neutral words. Thus, there was a facilitative effect for positive words and an inhibitory effect for negative words. The results of the categorical valence model support this finding (see Fig. 3). In addition, multiple comparisons in both models revealed significant differences between the three valence levels (all *p*s < .003). On the other hand, a facilitative effect of arousal was also observed, indicating that words with high arousal values were recognised faster than words with low arousal values, *β* = – 2.39, SE = 0.60, *t* = – 4.01, *p* < .001.

**Fig. 4:**
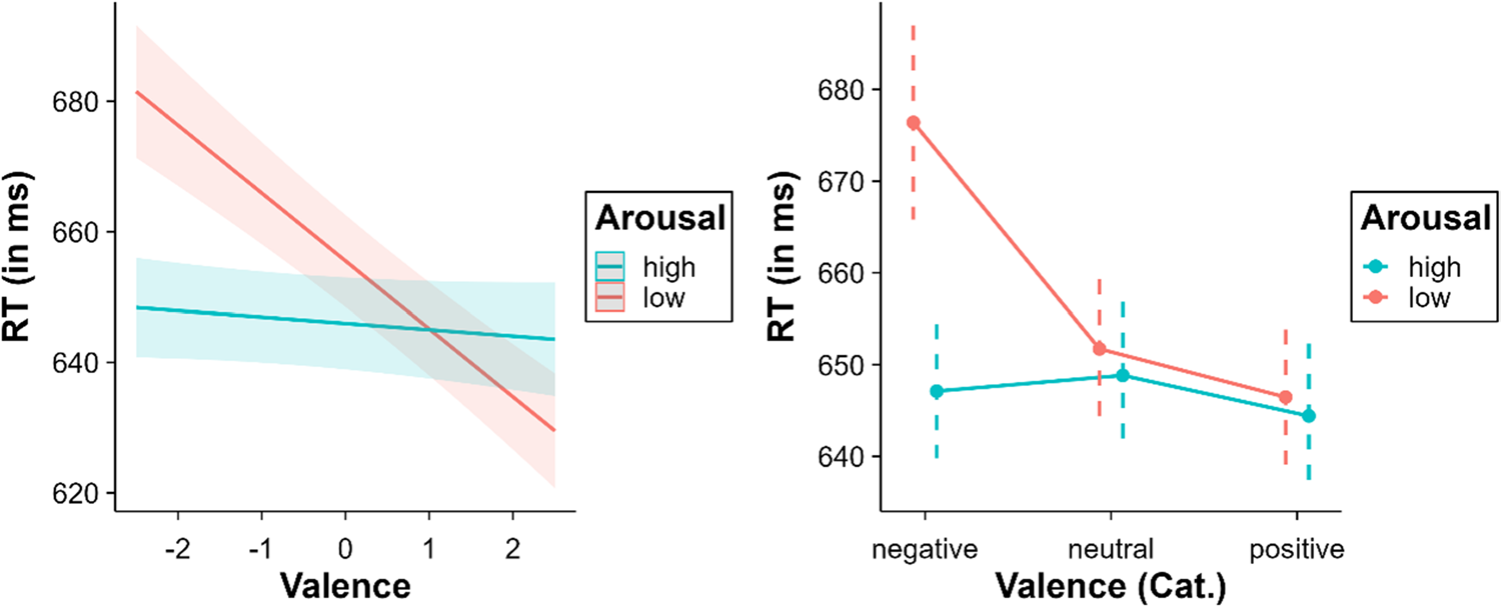
Interaction between valence and arousal (*left* = continuous valence model; *right *= categorical valence model). High and low levels of arousal correspond to +2 SD and – 2 SD, respectively, in relation to the mean arousal of the words

Importantly, the effects of valence and arousal were moderated by the interaction between both variables, *β* = 2.36, SE = 0.44, *t* = 5.35, *p* < .001 (see Fig. 4). Arousal affected negative and positive words differently. A facilitative effect was observed for negative words: High-arousal negative words were recognised faster than low-arousal negative words (in both the continuous and the categorical valence models, all *p*s < .001). The opposite effect was observed for positive words, that is, an inhibition for high arousal positive words relative to low arousal positive words (although this was only significant in the continuous valence model, *p* = .025).

### Valence x extroversion

**Fig. 5:**
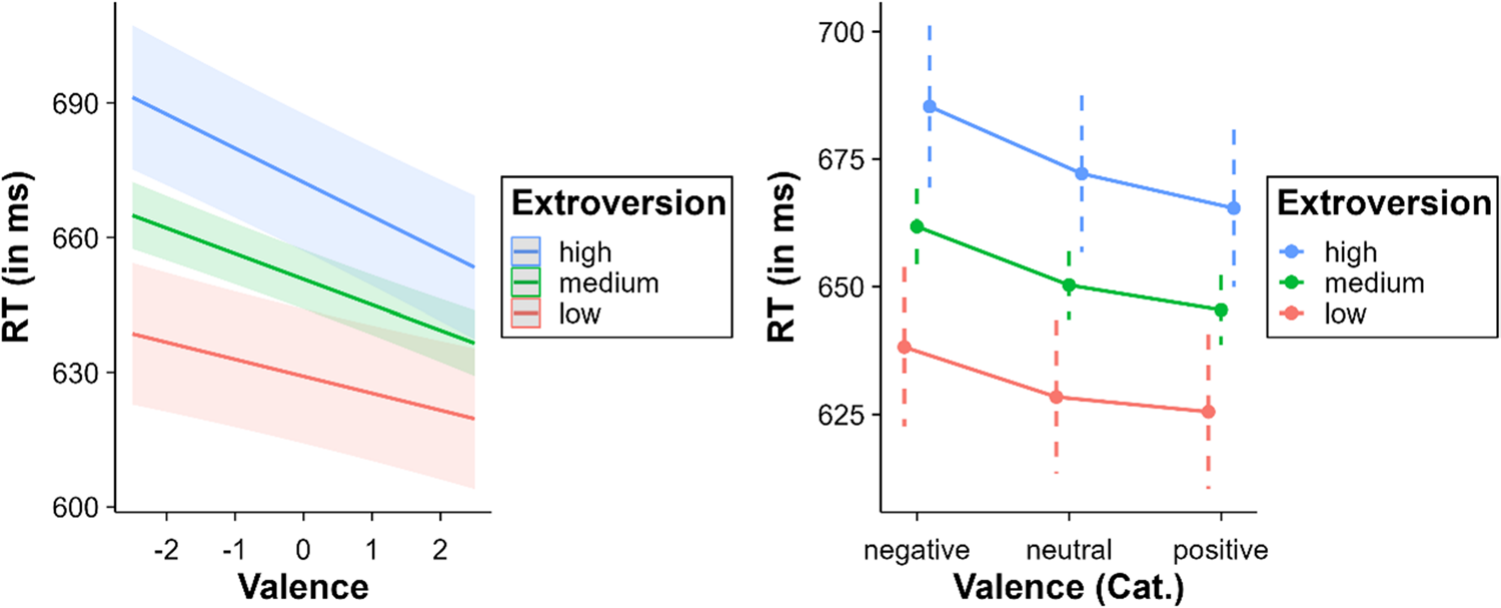
Interaction between valence and extroversion (*left* = continuous valence model; *right* = categorical valence model)

A significant interaction was found between valence and extroversion: *β* = – 0.95, SE = 0.36, *t* = – 2.63, *p* = .008. The effect of valence became larger as the level of extroversion increased (low extroversion = – 7.56 ms, medium extroversion = – 11.36 ms, high extroversion = – 15.16 ms; see Fig. 5). This increment in the valence effect was mostly due to differences in RT to positive words between the three levels of extroversion. Multiple comparisons from the categorical valence model revealed no differences between positive and neutral words at low levels of extroversion (*p* = .265), while a facilitation for positive words relative to neutral words was observed at medium and high levels of extroversion (both *p*s < .005). In contrast, negative words showed longer RT than neutral and positive words at all levels of extroversion (all *p*s < .002).

### Valence x conscientiousness

**Fig. 6:**
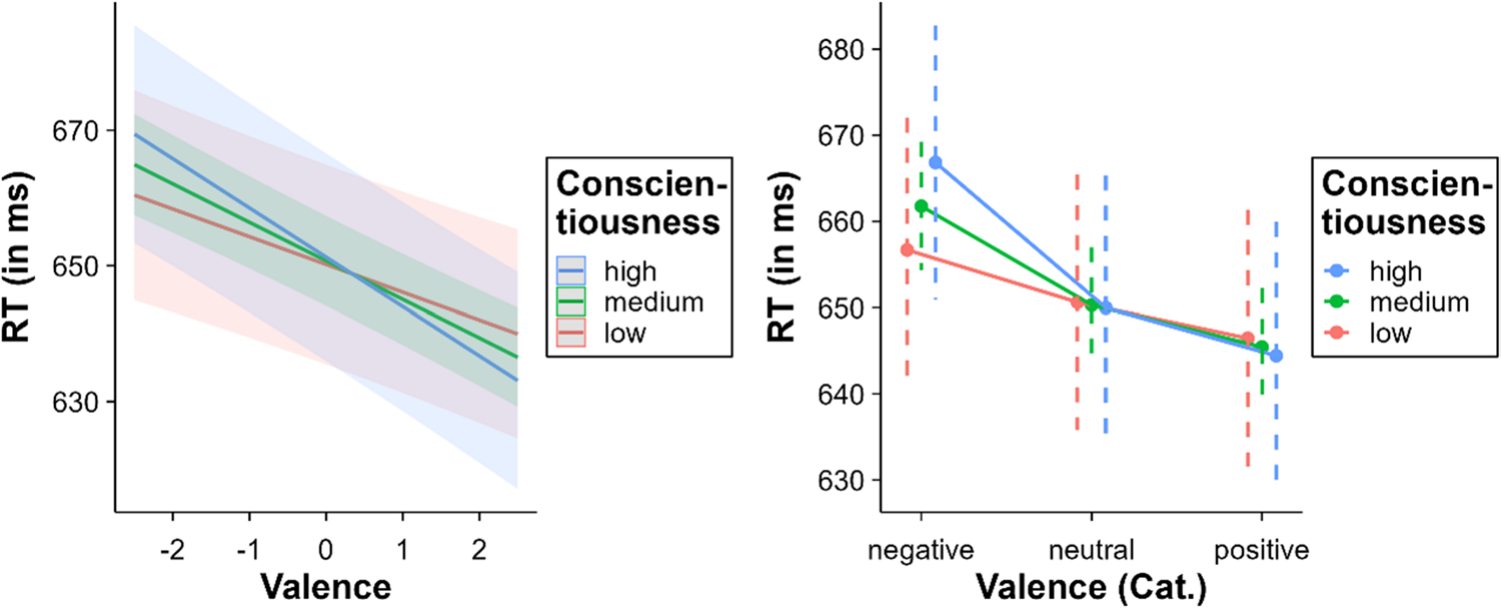
Interaction between valence and conscientiousness (*left* = continuous valence model; *right* = categorical valence model)

The interaction between valence and conscientiousness was significant, *β* = – 0.79, SE = 0.36, *t* = – 2.24, *p* = .025. The effect of valence increased along with the level of conscientiousness (low conscientiousness = – 8.18 ms, medium conscientiousness = – 11.36 ms, high conscientiousness = – 14.54 ms; see Fig. 6). Multiple comparisons within the categorical valence model showed no differences at low levels of conscientiousness either between negative and neutral words (*p* = .067) or between positive and neutral words (*p* = .061). In contrast, all the multiple comparisons between negative, neutral, and positive words were significant at medium and high levels of conscientiousness (all *p*s < .01).

### Valence x openness to experience and arousal x openness to experience

**Fig. 7:**
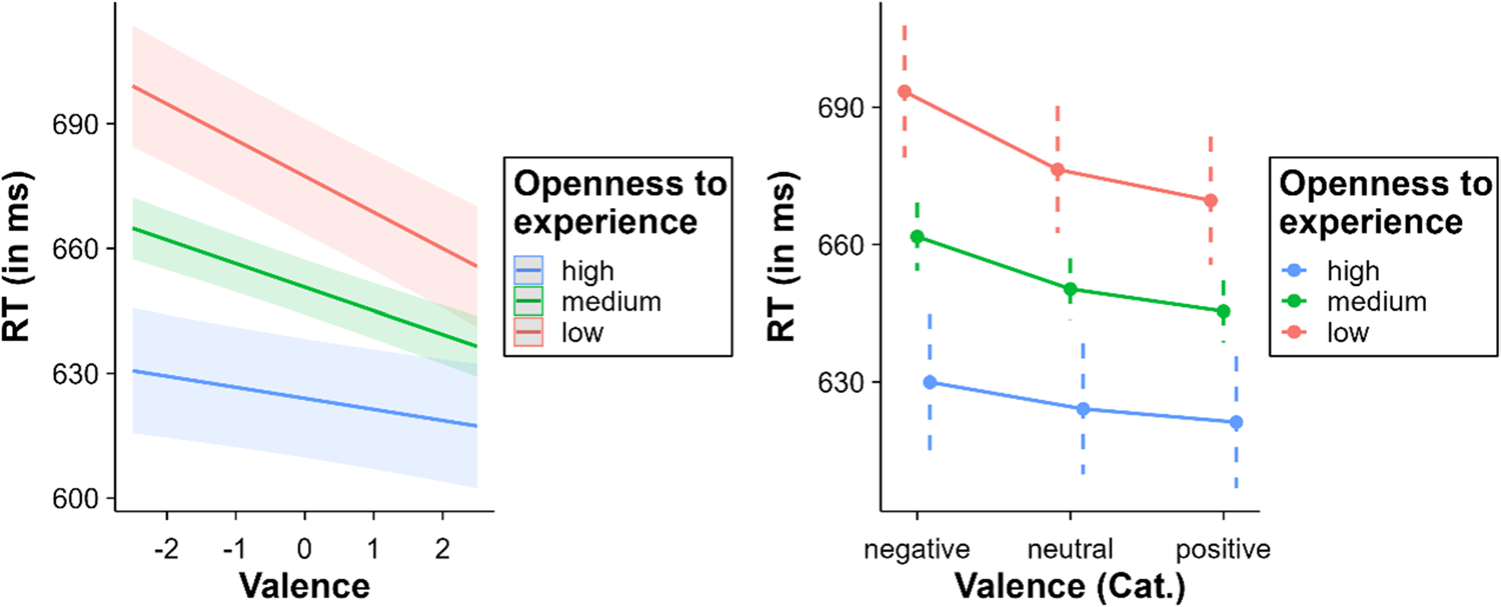
Interaction between valence and openness to experience (*left* = continuous valence model; *right* = categorical valence model)

**Fig. 8:**
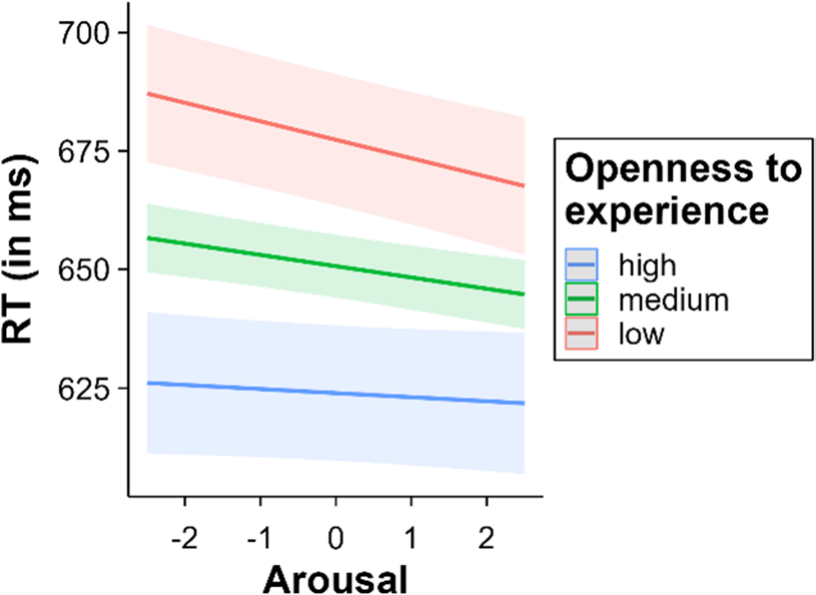
Interaction between arousal and openness to experience

Valence interacted significantly with openness to experience, *β* = 1.51, SE = 0.33, *t* = 4.55, *p* < .001. The effect of valence decreased as openness to experience increased (low openness to experience = – 17.38 ms, medium openness to experience = – 11.35 ms, high openness to experience = – 5.32 ms; see Fig. 7). Multiple comparisons within the categorical valence model in the high level of openness to experience showed neither significant differences between negative and neutral words (*p* = .075), nor between positive and neutral words (*p* = .24). Contrastingly, all comparisons between negative, neutral, and positive words were significant at medium and low levels of openness to experience (all *p*s < .001). Additionally, arousal also interacted with openness to experience, *β* = 0.76, SE = 0.33, *t* = 2.30, *p* = .021. It was in the same direction as valence: The higher the openness to experience, the lower the arousal effect, disappearing at the highest level (low openness to experience = – 7.81 ms, medium openness to experience = – 4.76 ms, high openness to experience = – 1.72 ms; see Fig. 8). Multiple comparisons showed significant differences between arousal levels in the medium and low openness to experience groups (all *p*s < .001). However, no differences were observed between different arousal levels in the high openness to experience group (all *p*s > .60).

### Valence x age and arousal x age

**Fig. 9:**
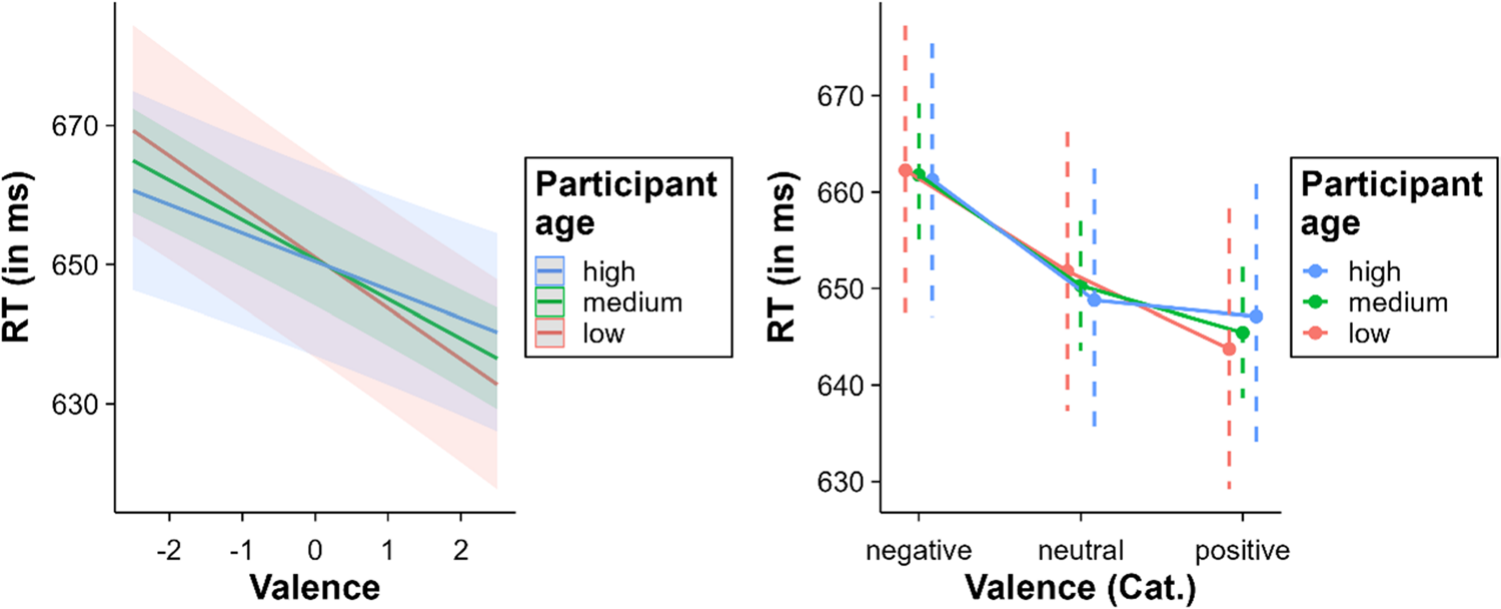
Interaction between valence and age (*left* = continuous valence model; *right* = categorical valence model)

**Fig. 10:**
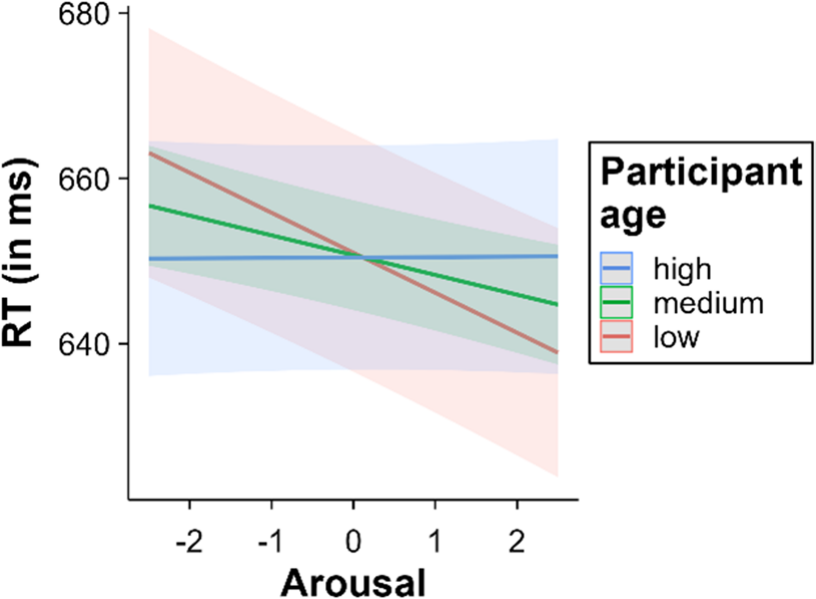
Interaction between arousal and age

The interaction between valence and age was significant, *β* = 0.81, SE = 0.33, *t* = 2.45, *p* = .014. The valence effect decreased with age (younger participants = – 14.60 ms, medium-age participants = – 11.37 ms, older participants = – 8.15 ms). This attenuation seems to be due to a lack of facilitation for positive words in older participants (see Fig. 9). Indeed, multiple comparisons within the categorical valence model showed no differences between positive and neutral words in older participants (*p* = .61). All other comparisons were significant at all age levels (all *p*s < .001). Furthermore, age interacted significantly with arousal, *β* = 1.22, SE = 0.33, *t* = 3.69, *p* < .001. Like in the case of valence, the arousal effect decreased with age (younger participants = – 9.67 ms, medium-age participants = – 4.79 ms, older participants = 0.10 ms; see Fig. 10), showing no significant effect in older participants (*p* = .99). In younger and middle-aged participants, arousal significantly decreased RT (both *p*s < .001).

### Valence x gender

**Fig. 11:**
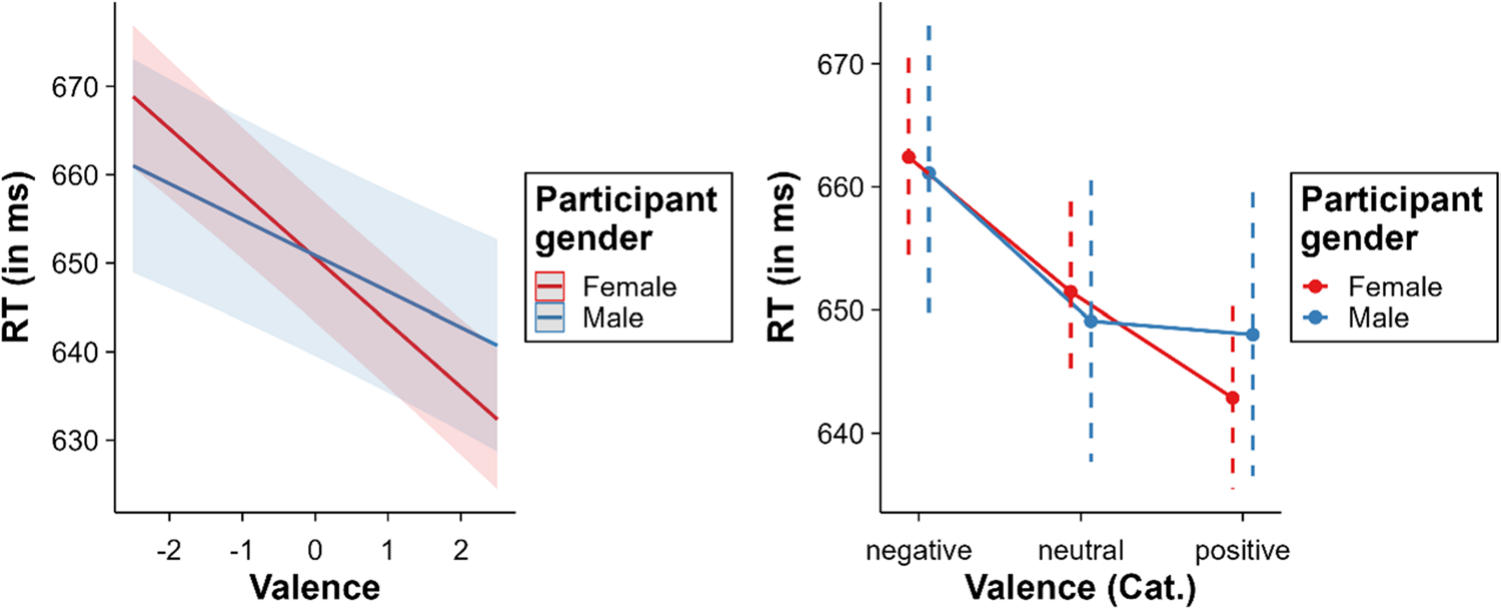
Interaction between valence and gender (*left* = continuous valence model; *right* = categorical valence model)

An interaction was found between valence and gender, *β* = 3.24, SE = 0.72, *t* = 4.49, *p* < 0.001. The effect of valence was larger for females (– 14.60 ms) than for males (– 8.12 ms) (see Fig. 11). The results from the categorical model suggest that the cause of this interaction is that males did not show significant differences between positive and neutral words (*p* = .767), whereas females did (*p* < .001). All other comparisons between valence levels and, importantly, the inhibition of negative words relative to neutral words (males: 12.01 ms, females: 10.95 ms) were significant for both males and females (all *p*s < .001).

## Discussion

This work presents an extensive lexical decision mega-study in Spanish. Our main objective was twofold: Firstly, to examine how emotional content influences word recognition, and the direction of the effect, while controlling for potential confounding variables; and secondly, to explore whether individual differences, such as personality traits, age, and gender modulate the effect.

The results showed a significant impact of both valence and arousal on the LDT, as well as an interaction between these two variables. The effect of valence was linear: RTs were slower as negative valence increased, and faster as positive valence increased. The advantage of positive valence is in line with several previous studies (e.g. Kuchinke et al., 2005; Siakaluk et al., 2016), while the inhibitory effect of negative valence is only consistent with part of the literature (e.g. Estes & Adelman, 2008; Larsen et al., 2008), as there are also reports of facilitative (e.g. Citron et al., 2013) and null (e.g. Larsen et al., 2006) effects. In any case, the linear valence effect found here mirrors the findings of a mega-study conducted in English by Kuperman et al. (2014), which included thousands of words and controlled for multiple confounding variables. The same pattern was obtained in a smaller study conducted in Spanish (Rodríguez-Ferreiro & Davies, 2019). In both cases, the findings were interpreted congruently with a graded version of the automatic vigilance hypothesis (Pratto & John, 1991), which posits that humans have an evolutionary tendency to prioritise negative stimuli, leading them to devote significant attention, consistently and automatically, to potential threats. Applied to word processing, this means that when the recognition system detects a word with negative emotional content, attention is quickly redirected and focused on that content. This attentional shift may interfere with ongoing cognitive tasks, such as deciding the lexicality of a word, thus delaying the recognition of negative words in the LDT (e.g. Estes & Adelman, 2008). Based on the linear valence effect found in our study and in Kuperman et al. and Rodriguez-Ferreiro and Davies, the impairment in word recognition seems to be proportional to the degree of threat posed by the emotional content: Words with more extreme negative valences would disrupt recognition to a greater extent, resulting in larger delays in lexical decision times.

The recognition advantage observed for positive words is probably rooted in a different mechanism. This facilitation is often related to the inherent positivity bias of language, which means that positive words are both more used and frequent than their negative and neutral counterparts (e.g. Dodds et al., 2015). This frequent use of positive words may lower their identification threshold. Consequently, these words would require less activation to be recognised (Kuperman et al., 2014). An alternative explanation relies on the observation that positive words seem to be more deeply connected and elaborated (Isen et al., 1985), more concrete, associated with richer sensory experiences, and have more meanings (Warriner et al., 2013), compared to neutral and negative words. This enhanced semantic richness may ultimately lead to faster recognition of positive words via semantic-to-orthographic feedback mechanisms (e.g. Pexman et al., 2008). This also suggests that valence effects, particularly those related to positive content, are semantically grounded. Such a notion propounds that the emotional content of a word is embedded in its semantic representation (e.g. Yap & Seow, 2014). Further research should explore this possibility to determine the exact nature of the influence of positive valence.

Noteworthily, valence effects interacted with arousal in this study. Although arousal had a general facilitating effect, the direction was opposite in positive and negative words: Arousal sped up the recognition of negative words but slowed that of positive words. This complex interplay between arousal and valence has been documented in several studies (e.g. Citron et al., 2014; Hoffman et al., 2009; Larsen et al., 2008; Vieitez et al., 2021) and is consistent with the model proposed by Robinson et al. (2004), according to which both negative valence and high arousal are associated with avoidance strategies, because they often signal threat. On the other hand, both positive valence and low arousal are seen as non-threatening or attractive situations and elicit approach strategies. Thus, different valence-arousal combinations can lead to either congruent (positive valence-low arousal, negative valence-high arousal) or incongruent (positive valence-high arousal, negative valence-low arousal) strategies, leading to a facilitated or a hindered word recognition, respectively.

The most novel contribution of this mega-study is related to its second aim, that is, to explore whether individual differences influence emotional word recognition. The evidence obtained here gives us a richer and more detailed understanding of such a process, making it clear that there is not a one-size-fits-all interpretation of the effects of emotional content on word recognition. Focusing on personality traits, extroversion levels modulated the valence effect. More extroverted participants showed a stronger valence facilitation effect compared to less extroverted counterparts. Importantly, this was not due to a general increase of the valence effect in more extroverted individuals; it seems rather that they showed a facilitation effect for positive words that was not seen in less extroverted participants. This observation is in line with previous behavioural findings obtained in the LDT (Borkenau et al., 2010), as well as with ERP data showing an association of higher extroversion levels with reduced N400 amplitudes for positive words relative to neutral words in the same task (Ku et al., 2020). Borkenau et al. (2010) suggested that extroverts may be more sensitive to positive emotional content, perhaps due to an accumulation of more positive life experiences in comparison with introverts. This predisposition could facilitate the activation and retrieval of emotional content associated with pleasant concepts. Similarly, Ku et al. (2020) considered that the N400 differences observed in their study indicate an advantage for extroverts in recognizing and conceptualizing the semantic content associated with positive words. This is also supported by language production studies showing that extrovert individuals tend to use more positive words compared to introvert individuals (Chen et al., 2020; Yarkoni, 2010). Finally, this influence of extroversion level on emotional valence effects also seems to be in line with reports showing the key role of affective experience in word representation (Ferré et al., 2023).

We also found an interaction between conscientiousness and valence. Mirroring the pattern observed for extroversion, the facilitating effect of valence became stronger as the level of conscientiousness increased: There was a significant valence effect in participants presenting average to high levels of conscientiousness, but not in participants with low levels of conscientiousness. A closer examination reveals that the effect was mainly due to a greater inhibition for negative words in more conscientious participants. Thus, although the relationship between conscientiousness and valence follows the same direction as that between valence and introversion, the reason is different, because it suggests a greater sensitivity to negative rather than to positive content in high conscientiousness individuals. This finding is consistent with the inverse relationship between conscientiousness and the use of negative words of (Caplan et al., 2020). A possibility is that highly conscientious individuals find it more difficult and time-consuming to retrieve and process the emotional content of negative words, given their infrequent use of these words. Considering the automatic vigilance hypothesis (Pratto & John, 1991), we might speculate that the increased cognitive load required to process infrequent negative words (for this variety of participants) leads to a stronger and more prolonged attentional shift, which may explain the greater inhibition in highly conscientious individuals relative to lowly conscientious individuals.

The interaction between openness to experience and valence reveals a different pattern compared to the above discussed interactions: Low openness individuals showed a greater valence facilitation. Notably, high openness individuals did not show a significant valence effect. The effect was particularly marked for negative words; as openness to experience increased, the inhibitory effect of negative words decreased and eventually disappeared at higher levels. It is the first time that this interaction has been reported in a psycholinguistic study. Therefore, hypotheses about this relationship should be tentatively grounded in broader research on how openness to experience influences behaviour. The literature suggests that individuals who score high in openness to experience tend to exhibit superior emotional regulation, often showing less avoidance and suppression behaviours (Barańczuk, 2019). This control over avoidance and suppression behaviours may influence the response of such individuals to threatening stimuli, giving them greater agency over how attention is allocated and maintained when they process negative emotional content. In terms of the automatic vigilance hypothesis (Pratto & John, 1991), this would reduce the influence of attentional shifting to negative words, consequently attenuating the delay typically observed with these words.

In addition to personality effects, our study also revealed some differences in emotional word recognition associated with participants' age and gender. Firstly, the influence of both valence and arousal appeared to decrease or disappear with age, particularly as the facilitation for positive words seen in younger participants was absent in the older cohort. At first glance, this finding seems to contradict previous reports showing that positivity bias increases with age, as demonstrated in word rating (e.g. Carstensen & DeLiema) and LDT studies (e.g. Ku et al., 2022), in which older individuals tend to rate words more positively and show a preferential processing of positive over negative information. However, the present results are consistent with those of Kyröläinen et al. (2021), who relied on the data of three large-scale LDT studies and found that the age-related positivity bias may not be as pervasive as once thought. Indeed, they observed an opposite trend: Older participants showed a reduced advantage for positive words compared to their younger counterparts. According to the authors, this could be explained by the "lifelong learning" hypothesis, which posits that as individuals age, their repertoire of positive words expands, leading to a larger but less distinctive set. Consequently, the distinctiveness of 'positivity' as an attribute to aid recognition diminishes over time as the pool of positive words increases. Of note, there is an important limitation that should be taken into account when interpreting the interaction of valence by age found here. The sample of the present study consisted mainly of young people, with a rather low proportion of old people. In fact, 74% of the participants were under 30 years of age and only 7% were 50 years old or older. Further studies offering a more representative sampling across age groups are needed to examine this interaction.

Finally, our results also reveal gender differences regarding the influence of emotional valence. Women showed a stronger valence effect compared to men. This is consistent with the study of Abbassi et al. (2019), in which women showed a higher facilitated emotional word recognition with respect to men. Our findings are also in line with the observation that women tend to give more extreme valence ratings to both positive and negative words than men (Soares et al., 2012; Söderholm et al., 2013). Taken together, these findings suggest a greater sensitivity to emotional valence in women and they contribute to refining this idea, showing that the gender effect is due to a lack of facilitation for positive words among male participants. Therefore, this heightened sensitivity in women may not be general for emotional information, but rather associated with positive content. In any case, this is a hypothesis to be put to the test in future studies.

In conclusion, this large-scale lexical decision study highlights the important role of emotional content in word recognition. In accordance with previous research, the results reveal a differential effect of emotional valence polarity: negative valence inhibits recognition, whereas positive valence facilitates it. Furthermore, the influence of positive and negative valence appears to be modulated by emotional arousal, suggesting that different combinations of these variables can elicit avoidance or approach strategies. Importantly, the present study is unique in extending the scope of emotional word recognition research by exploring individual difference effects via a large sample of participants and words. We provide some novel evidence on how individual differences affect emotional word recognition. In particular, we found that emotional valence effects are influenced by personality traits (extroversion, conscientiousness, and openness to experience), age, and gender. This suggests that the processing of emotional word content is not uniform across the population. However, a limitation of the study must be acknowledged. Namely, despite the sample’s diversity in terms of background, age, and gender, there was some bias in the gender ratio (70% female, 30% male) and also in its age distribution. Future research should attempt to test more balanced samples. Finally, in addition to the contribution of this mega-study to our knowledge of emotional word recognition and its modulation by individual differences, it also provides valuable data for researchers in the field: We provide a large database containing RTs for 7500 Spanish words from 918 participants, as well as values for individual differences (personality, age, and gender) and several lexical, semantic and emotional variables. Although most of the psycholinguistic values used here come from various published databases, we also provide values for concreteness (*n* = 1690), familiarity (*n* = 1693) and age of acquisition (*n* = 2171), which were collected exclusively for this study.

## Data Availability

The data obtained in this study can be downloaded from the following online open-access repository (Open Science Foundation): https://osf.io/cbtqy

## Footnotes

1 Analyses of the RTs using the inverse transformation (– 1000/RT), or the logarithmic transformation showed similar results.

2 In another set of analyses, we used the same procedure as Kuperman et al. (2014) to reduce multicollinearity by grouping lexical variables into principal components. The results were almost identical to those reported here (see “Appendix C” in the OSF repository).

## Code availability

Code will be made available on request.
